# Factors associated with use of breast cancer screening services by women aged ≥ 40 years in Korea: The Third Korea National Health and Nutrition Examination Survey 2005 (KNHANES III)

**DOI:** 10.1186/1471-2407-10-144

**Published:** 2010-04-16

**Authors:** Kiheon Lee, Hyung Taek Lim, Sang Min Park

**Affiliations:** 1 Department of Family Medicine, Seoul National University Bundang Hospital, 166 Gumi-ro, Bundang-gu, Seongnam-si Gyeonggi-do, 463707, Korea; 2 Department of the History of Medicine and Medical Humanities, Seoul National University College of Medicine, 101 Daehangno, Jongno-gu, Seoul, 110744, Korea; 3 Department of Ophthalmology, Yonsei University College of Medicine, 134 Shinchon-dong, Seodaemun-gu, Seoul, 120752, Korea; 4 Department of Family Medicine, Seoul National University Hospital, Seoul National University College of Medicine, 101 Daehangno, Jongno-gu, Seoul, 110744, Korea

## Abstract

**Background:**

Despite evidence that breast cancer screening reduces morbidity and mortality, until recently most women have not undergone regular mammogram examinations in Korea. We aimed to identify factors associated with use of breast cancer screening services.

**Methods:**

The Health Promotion Knowledge, Attitude and Practice survey (HP-KAP survey) is part of the Third Korea National Health and Nutrition Examination Survey 2005 (KNHANES III), a nationwide health survey in Korea. Of 7,802 individuals who participated in the HP-KAP survey, 4,292 were female. Of these, 2,583 were women aged at least 40 years and without a history of breast cancer; these women were included in this study. Information about breast cancer screening participation was obtained from the responses to questionnaires. The overall rate of regular breast cancer screening was measured. Factors that affect participation in a breast cancer screening program were identified using multiple logistic regression analysis.

**Results:**

Among women aged at least 40 years, 30.4% complied with breast screening recommendations. Age of at least 65 years (adjusted odds ratio, aOR 0.61, 95% CI: 0.42-0.88), education level (no [ref], elementary school [aOR 1.51, 95% CI: 1.06-1.47], middle/high school [aOR 1.99, 95% CI: 1.36-2.92], university/higher [aOR 2.73, 95% CI: 1.71-4.35]), private health insurance (aOR 1.42, 95% CI: 1.71-4.35), attitude towards screening tests (aOR 0.18, 95% CI: 0.14-0.23), self-reported health status of 'fair' (aOR 1.26 95% CI: 1.00-1.58), and smoking (aOR 0.52, 95% CI: 0.35-0.79) were associated with the rate of regular breast cancer screening

**Conclusions:**

To increase the nationwide breast cancer screening rate, more attention should be given to underrepresented groups, particularly the elderly, those with a low education level, smokers, and those with a negative attitude towards screening tests. These issues highlight the need for a new emphasis in health education, promotional campaigns and public health policy aimed at these underrepresented groups.

## Background

Breast cancer is the most common female cancer, with more than 1 million cases and nearly 600,000 deaths occurring worldwide annually [1]. In Korea, the incidence of breast cancer in 2006 was 46 per 100,000 women; previous data have been reported elsewhere [2-5]. Epidemiological factors indicate that the incidence and mortality rate of breast cancer will increase in Korea [6,7].

Mammography screening is known to reduce mortality from breast cancer [8-10], based on results from at least nine major randomized controlled trials [11]. Korea has an organized population-based screening program in which almost all Korean women aged 40 years or more regularly receive a personal letter inviting them to undergo breast cancer screening. In 1996, the Korea National Cancer Screening Program (KNCSP) recommended that all Korean women aged 40 years or more undergo mammography examinations every 1-2 years to screen for breast cancer. In 1999, KNCSP became responsible for the provision of free screening services for low-income, Medicaid recipients. Since then, the KNCSP has expanded its target population to include all National Health Insurance (NHI) beneficiaries. Currently, KNCSP provides the Medical Aid recipients and NHI beneficiaries within the lower 50% income bracket with free screening services for breast cancer. In case of NHI beneficiaries within the upper 50% income bracket, the NHI covers 80% of the cost and the beneficiary pays the remaining 20%. Despite evidence that breast cancer screening reduces morbidity and mortality, until recently most Korean women have not undergone regular mammogram examinations (10-50%) [12,13]. Therefore, to increase the participation rate and improve the survival rate of breast cancer patients, identification and removal of potential barriers to cancer screening participation might be of great importance. However, individual and environmental circumstances that might independently contribute to the rate of participation in screening programs have not been thoroughly studied in Asian populations.

**Figure 1:**
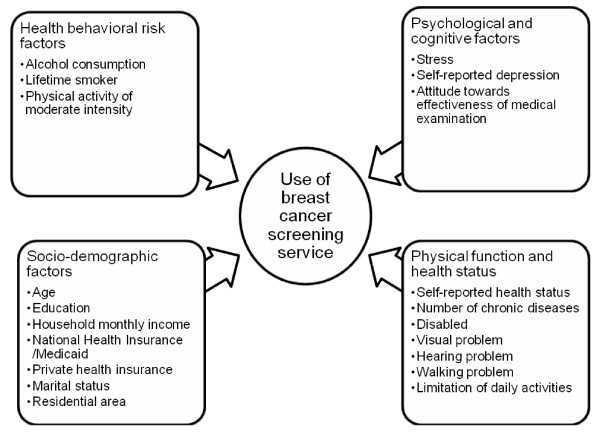
**Framework for this study**.

In this study, we investigated how socio-demographic factors, health behavioral risk factors, psychological and cognitive factors, and physical function and health status affect participation in breast cancer screening programs (Figure 1) among members enrolled in a large health survey in Korea; The Third Korea National Health and Nutrition Examination Survey 2005 (KNHANES III).

## Methods

### Subject selection

**Figure 2:**
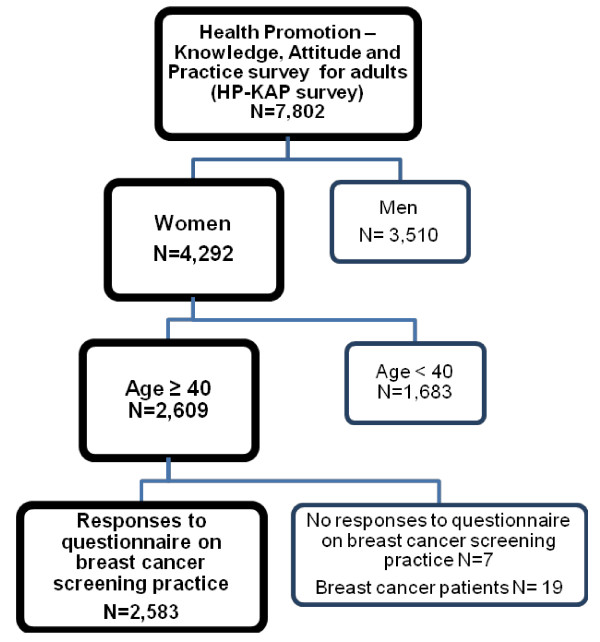
**Flow diagrams showing selection of the study population**.

The third (2005) KNHANES is a national health survey in Korea that involves population-based random sampling of 34,145 individuals in households across 600 national districts. A stratified multistage probability sampling design was used. To assure the equal probability of being sampled, weightings were assigned to each respondent. The third (2005) KNHANES is composed of four parts: the Health Interview survey, the Health Examination survey, the Nutrition survey and the Health Promotion Knowledge, Attitude and Practice (HP-KAP) survey. As the item about breast cancer screening was included in HP-KAP survey, we started with cross-sectional data from the HP-KAP survey. HP-KAP survey was conducted in each household as face-to-face interviews by trained interviewers. In the third (2005) KNHANES, 8,417 individuals aged ≥ 19 year were sampled as subjects of the HP-KAP survey. Among them, 7,802 individuals participated in the examination: the response rate was 92.7%. Figure 2 shows the model used to select our study population. Subjects of male gender, aged less than 40 years, whose responses were incomplete, or who had a prior diagnosis of breast cancer were excluded from the study, leaving a total of 2,583 subjects. As the survey data analyzed are publicly available, ethical approval was not needed for this study

### Breast cancer screening outcome measures

Subjects were asked the question "when was the last time you had a mammography or ultrasonography by a doctor?" with possible responses of "never", "≤ 2 years ago", and ">2 years ago". This question was designed based on screening recommendations from the KNCSP, which recommend breast cancer screening by mammogram or clinical breast examination (CBE) for every woman aged ≥ 40 years at 2-year intervals [14]. Subjects who had undergone a mammography or breast ultrasonography examination no more than 2 years ago were considered as having undergone breast cancer screening as outlined by the KNCSP guidelines. Data were collected using a self-administered questionnaire.

### Independent variables

From KNHANES III, we collected information about various factors associated with breast screening. We assessed variables in relation to socio-demographic factors, health behavioral risk factors, psychological and cognitive factors, and physical function and health status. The socio-demographic variables were: current age (40-49/50-64/≥ 65 years), highest educational level reached (no education/elementary school graduates/middle or high school graduates/university or higher graduates), household monthly income (<650/650-1,345/≥ 1,345 $US), having NHI or Medicaid (NHI/Medicaid), having supplementary private health insurance (PHI) (yes/no), marital status (living with spouse/living without a spouse) and residential area (urban/rural). The health behavioral risk variables included alcohol consumption (never/less than once in a month/more than once in a month), lifetime smoker (no/yes), and regular physical activity of moderate intensity (never/more than once in a week/everyday). The psychological and cognitive variables were feeling stress (often/rarely), self-reported depression (yes/no) and attitude to the effectiveness of preventive medical evaluations (effective/not effective or not having received a medical examination). The physical function and health status variables were self-reported general health status (healthy/fair/unhealthy), registered as a disabled person (yes/no), number of chronic diseases (0-3/≥ 4), visual problems (no problem/yes), hearing problems (no problem/yes), walking problems (no problem/yes), and limitation in daily activities (no limitation/yes). Data were collected using a self-administered questionnaire.

Household monthly income was divided into tertiles. Income per adult equivalent was calculated using the formula household income/square root of number of persons in the household [15]. The term 'spouse' was applied to individuals who are legally married or cohabiting, the term 'without spouse' was applied to single, divorced, or separated individuals. All respondents were asked if they had smoked a total of 100 cigarettes in their life [16]. Lifetime smoker included respondents who reported that they have smoked at least 100 cigarettes in their lifetime and now smoke. Non-smoker included respondents who have smoked less than 100 cigarettes in their lifetime but currently do not smoke.

We defined moderate-intensity activities as those lasting at least 10 minutes and which increased the individual's heart rate slightly compared with sedentary activities; examples included volleyball, table tennis, swimming, yoga, and badminton, but walking was not included [17].

Patients who had a history of chronic diseases (such as hypertension, diabetes, cardiovascular disease, lung disease, musculoskeletal disease, gastrointestinal disease, and anemia) were classed as one of two groups, those who had experienced 0-3 chronic diseases and those who had ≥ 4 chronic diseases.

### Statistical methods

Descriptive statistics were reported for each response. We used a two-step, multi-dimensional approach to identify factors predictive of breast screening. First, to identify the factors associated with participation in a breast screening program, odds ratios for attendance and 95% confidence intervals were calculated by univariate logistic regression analysis. Second, multiple logistic regression analysis was used to identify significant associated factors with use of breast cancer screening services. All factors identified as affecting participation in a breast cancer screening program by univariate analysis were included in the multivariate analysis with enter method. All statistical tests were two-sided at 95% confidence intervals and performed using STATA 10.0 (StataCorp, College Station, Texas, USA)

## Results and Discussion

### Study population baseline characteristics

The mean age of the 2,583 women included in our study was 55.89 years; 65.49% had NHI and PHI, 68.49% were living with a spouse, 75.57% were living in urban areas, and 90.09% were nonsmokers. Table 1 shows the baseline characteristics of study participants. Among women aged ≥ 40 years, the compliance with breast screening recommendations was 30.43%.

**Table 1:** Characteristics of the study population (n = 2,583)

Socio-demographic factors		n	%
Age (years)	40-49	1001	38.75
	50-64	922	35.69
	65+	660	25.55
Education	No	422	16.34
	Elementary school (≤ 6 years)	751	29.07
	Middle/high school (7-12 years)	1146	44.37
	University/higher (≥ 13 years)	264	10.22
Household monthly income^1^	Lowest tertile (≤ US$650)	851	32.95
	Middle tertile (US$650-1,345)	866	33.53
	Highest tertile (≥ US$1,345)	835	32.33
National health insurance (NHI)/Medicaid	NHI	2428	94.00
	Medicaid	155	6.00
Private health insurance (PHI)	No	948	36.70
	Yes	1629	63.07
NHI with or without PHI	NHI with PHI	1590	65.49
	NHI without PHI	832	34.27
Medicaid with or without PHI	Medicaid with PHI	39	25.16
	Medicaid without PHI	116	74.84
Marital status^2^	With spouse	1769	68.49
	Without spouse	812	31.44
Residential area	Urban	1952	75.57
	Rural	631	24.43
**Health behavioral risk factors**			
Alcohol	Never	1062	41.11
	Less than once per month	795	30.78
	More than once per month	726	28.11
Lifetime smoker^3^	No	2348	90.90
	Yes	235	9.10
Physical activity of moderate intensity^4^	Never	1558	60.32
	More than once per week	719	27.84
	Everyday	306	11.85
**Psychological and cognition factors**			
Stress	Often	926	35.85
	Rarely	1657	64.15
Self-reported depression^5^	No	2036	78.82
	Yes	546	21.14
Attitude towards effectiveness of medical examination	Effective	1589	61.52
	Not effective/not received medical exam.	993	38.44
**Physical function and health status**			
Self-reported health status	Healthy	693	26.83
	Fair	947	36.66
	Unhealthy	943	36.51
Number of chronic diseases^6^	0-3	1497	57.96
	4+	1086	42.04
Disabled^7^	No	2494	96.55
	Yes	89	3.45
Visual problem	No	1544	59.78
	Yes	1039	40.22
Hearing problem	No	2202	85.25
	Yes	381	14.75
Walking problem	No	1931	74.76
	Yes	652	25.24
Limitation in daily activities	No	2136	82.69
	Yes	447	17.31

^1 ^To calculate income per adult equivalent, we defined as household 'income/square root of person'

^2 ^The term 'spouse' refers to an individual who is legally married or cohabiting, 'without spouse' refers to an individual who is single, divorced, or separated

^3 ^'Lifetime smoker' included respondents who reported that they have smoked at least 100 cigarettes in their lifetime and now smoke

^4 ^Moderate-intensity activitieswere defined as lasting at least 10 minutes and increasing heart rate slightly compared with sedentary activities

^5 ^Experience of depression was assessed by the question, "During the past 1 year, have you often been bothered by little interest or pleasure in doing things nearly every day for the past 2 weeks?"

^6 ^Chronic diseases such as hypertension, diabetes, cardiovascular disease, lung disease, musculoskeletal disease, gastrointestinal disease, and anemia

^7 ^Registered as a disabled person

### Factors associated with breast cancer screening practices

The factors shown to be associated with breast screening by univariate analysis (reported as odds ratios) were age, education level, household monthly income, PHI status, marital status, alcohol consumption, smoking status, physical activity level, attitude towards effectiveness of medical examination, self-reported health status, visual problem, hearing problem, walking problem, and limitation in daily activities (Table 2). When the variables identified as important by univariate analysis were combined in a multiple logistic regression analysis, only six of the factors were shown to be significant (Table 3). Women aged ≥ 65 years (adjusted odds ratio [aOR] = 0.61; 95% CI, 0.42-0.88) were less likely to undergo breast screening compared with those in the reference category (40-49 years). Women who had graduated from elementary school (aOR = 1.51; 95% CI, 1.06-2.16), middle/high school (aOR = 1.99; 95% CI, 1.36-2.92), or university or other higher education institute (aOR = 2.73; 95% CI, 1.71-4.35) were more likely to undergo breast cancer screening compared with women who had received no formal education. Based on the aOR, women with PHI were more likely to undergo breast screening compared with those without PHI (aOR = 1.42; 95% CI, 1.12-1.79). We observed an approximately two-fold decrease in breast screening among smokers compared with nonsmokers (aOR = 0.52; 95% CI, 0.35-0.79). Women with a positive attitude towards the effectiveness of medical examinations were also more likely to undergo breast screening compared with women with a negative attitude or those who had not previously undergone a medical examination (aOR = 0.18; 95% CI, 0.14-0.23). Breast cancer screening was also more common in women with a self-reported health status of 'fair' (aOR = 1.26; 95% CI, 1.00-1.58).

**Table 2:** Factors associated with breast cancer screening practice^1 ^in univariate analysis (n = 2,583)

Socio-demographic factors		%	univariate OR	95%CI	
Age (years)	40-49	35.26	1.0(ref)		
	50-64	35.25	1	0.83	1.21
	65+	16.36	0.36	0.28	0.46
Education	No	14.69	1.0(ref)		
	Elementary school (≤ 6 years)	26.90	2.14	1.56	2.92
	Middle/high school (7-12 years)	35.17	3.15	2.34	4.23
	University/higher (≥ 13 years)	45.08	4.77	3.32	6.85
Household monthly income^2^	Lowest tertile (≤ US$650)	22.68	1.0(ref)		
	Middle tertile (US$650-1,345)	30.37	1.49	1.2	1.85
	Highest tertile (≥ US$1,345)	37.96	2.09	1.69	2.58
National health Insurance (NHI)/Medicaid	NHI	30.6			
	Medicaid	27.74	0.87	0.61	1.25
Private health insurance	No	20.57	1.0(ref)		
	Yes	36.16	2.19	1.81	2.64
Marital status^3^	With spouse	33.69	1.0(ref)		
	Without spouse	23.4	0.6	0.5	0.73
Residential area	Urban	31.3	1.0(ref)		
	Rural	27.73	0.84	0.69	1.03
**Health behavioral risk factors**					
Alcohol	Never	27.5	1.0(ref)		
	Less than once per month	33.96	1.36	1.11	1.66
	More than once per month	30.85	1.18	0.96	1.45
Lifetime smoker^4^	No	31.98	1.0(ref)		
	Yes	14.89	0.37	0.26	0.54
Physical activity of moderate intensity^5^	Never	27.34			
	More than once per week	36.44	1.52	1.26	1.84
	Everyday	32.03	1.25	0.96	1.63
**Psychological and cognitive factors**					
Stress	Often	29.05	1.0(ref)		
	Rarely	31.2	1.11	0.93	1.32
Depression^6^	No	30.7	1.0(ref)		
	Yes	29.49	0.94	0.77	1.16
Attitude towards effectiveness of medical examination	Effective	42.42	1.0(ref)		
	Not effective/not received medical exam.	11.28	0.17	0.14	0.22
**Physical function and health status**					
Self-reported health status	Healthy	32.61	1.0(ref)		
	Fair	34.64	1.09	0.89	1.35
	Unhealthy	24.6	0.67	0.54	0.84
Number of chronic diseases^7^	0-3	31.73	1.0(ref)		
	4+	28.64	0.86	0.73	1.02
Disabled^8^	No	30.55	1.0(ref)		
	Yes	26.97	0.84	0.52	1.35
Visual problem	No	32.12	1.0(ref)		
	Yes	27.91	0.82	0.69	0.97
Hearing problem	No	31.65	1.0(ref)		
	Yes	23.36	0.66	0.51	0.85
Walking problem	No	32.63	1.0(ref)		
	Yes	23.93	0.65	0.53	0.8
Limitation in daily activities	No	31.79	1.0(ref)		
	Yes	23.94	0.68	0.53	0.85

^1 ^Subjects who had undergone a mammography or breast ultrasonography within the previous 2 years were considered as having received breast cancer screening as recommended by the KNCSP guidelines

^2 ^To derive income per adult equivalent, we defined as household 'income/square root of person'

^3 ^The term 'spouse' refers to an individual who is legally married or cohabiting, 'without spouse' refers to an individual who is single, divorced, or separated

^4 ^'Lifetime smoker' included respondents who reported that they have smoked at least 100 cigarettes and still smoke

^5 ^Moderate-intensity activitiesas were defined as lasting at least 10 minutes and increasing heart rate slightly compared with sedentary activities

^6 ^Experience of depression was assessed by the question, "During the past 1 year, have you often been bothered by little interest or pleasure in doing things nearly every day for the past 2 weeks?"

^7 ^Chronic diseases such as hypertension, diabetes, cardiovascular disease, lung disease, musculoskeletal disease, gastrointestinal disease, and anemia

^8 ^Registered as a disabled person

**Table 3:** Factors associated with breast cancer screening practice^1 ^in multivariate analysis^2 ^(n = 2,583)

Socio-demographic factors		Multivariate OR	95%CI	
Age (years)	40-49	1.0(ref)		
	50-64	1.16	0.92	1.47
	65+	0.61	0.42	0.88
Education	No	1.0(ref)		
	Elementary school (≤ 6 years)	1.51	1.06	2.16
	Middle/high school (7-12 years)	1.99	1.36	2.92
	University or higher (≥ 13 years)	2.73	1.71	4.35
Household monthly income^3^	Lowest tertile (≤ US$650)	1.0(ref)		
	Middle tertile (US$650-1,345)	0.94	0.73	1.21
	Highest tertile (≥ US$1,345)	0.99	0.75	1.3
Private health insurance	No	1.0(ref)		
	Yes	1.42	1.12	1.79
Marital status^4^	With spouse	1.0(ref)		
	Without spouse	1.07	0.85	1.35
**Health behavioral risk factors**				
Alcohol	Never	1.0(ref)		
	Less than once per month	1.09	0.87	1.37
	More than once per month	1.00	0.78	1.27
Lifetime smoker^5^	No	1.0(ref)		
	Yes	0.52	0.35	0.79
Physical activity of moderate intensity^6^	Never	1.0(ref)		
	More than once per week	1.11	0.9	1.37
	Everyday	1.12	0.84	1.5
**Psychological and cognitive factors**				
Attitude towards effectiveness of medical examination	Effective	1.0(ref)		
	Not effective/not received medical exam	0.18	0.14	0.23
**Physical function and health status**				
Self-reported health status	Healthy	1.0(ref)		
	Fair	1.26	1.00	1.58
	Unhealthy	0.94	0.71	1.24
Visual problem	No	1.0(ref)		
	Yes	1.05	0.86	1.29
Hearing problem	No	1.0(ref)		
	Yes	0.96	0.71	1.3
Walking problem	No	1.0(ref)		
	Yes	1.21	0.91	1.62
Limitation in daily activities	No	1.0(ref)		
	Yes	1.15	0.84	1.58

^1 ^Subjects who had undergone a mammography or breast ultrasonography within the previous 2 years were considered as having received breast cancer screening as recommended by the KNCSP guidelines

^2 ^Based on a multiple regression model including variables which were significantly associated with breast cancer screening participation in univariate analysis

^3 ^To derive income per adult equivalent, we defined as household 'income/square root of person'

^4 ^The term 'spouse' refers to an individual who is legally married or cohabiting, 'without spouse' refers to an individual who is single, divorced, or separated

^5 ^'Lifetime smoker' included respondents who reported that they have smoked at least 100 cigarettes and still smoke

^6 ^Moderate-intensity activitieswere defined as lasting at least 10 minutes and increasing heart rate slightly compared with sedentary activities

The data from the current study indicate that participation in breast cancer screening programs is less than optimal among Korean women aged ≥ 40 years. In addition, we found that advanced age, low education level, smoking, and a negative attitude towards preventive medical examinations were significantly associated with poor participation in breast cancer screening programs. As national survey with representative sample was used in our study, this strengthens generalizability of our results and could provide a nationwide surveillance assessment of under-utilization of breast cancer screening.

Advanced age is widely known as a risk factor for breast cancer and the importance of breast cancer screening in the elderly has been highlighted for many years[18,19]; this is why the KNSP recommends that all Korean women aged ≥ 40 years, including those aged ≥ 65 years, undergo breast cancer screening every 2 years. Similar to previous studies that investigated socio-demographic factors [20], we found a negative correlation between program participation and age. A previous study has shown that use of a 'reminder' system (web proactive system) can improve mammography rates [21]. Although methods to improve the mammography rates in elderly women have not been well studied, some studies have indicated that increased knowledge of breast cancer screening and free mammography examinations affect breast screening rates in the elderly [22,23] It is well known that, for elderly individuals, having a primary physician and making regular visits to this healthcare provider can increase medical screening rates [24]; hence, physicians should be encouraged to recommend that women undergo breast cancer screening.

Our results indicate that, in Korea, household monthly income is not significantly associated with the breast screening rate, whereas there is a large disparity in mammography use among women of different education levels. Other studies have used multivariate logistic regression analysis to show that women were more likely to undergo a mammography examination if they had a higher education level. However, some studies have suggested that women with an average level of education were more likely to participate in organized screening programs [20,25,26]. Despite this, a general positive correlation between education level and breast cancer screening program participation seems likely. Other studies have suggested that household income affects breast cancer screening program participation, with women from low-income households less likely to participate than those from high-income households [27,28]; however, this has not been reported in all studies [29,30]. A possible cause of this difference is that, in Korea, all individuals are entitled to NHI and the government pays 50% of the mammography examination fee. In addition, through the KNCSP, the Korean government has provided free screening services for individuals on low incomes and those receiving Medicaid since 1999. Such government financial support might have reduced the effects of household monthly income on breast screening participation.

In Korea, basic medical expenses are covered by the NHI and PHI is used as a supplement only by those individuals who require additional medical cover [31]; for example, those who experience a heart attack, stroke, or cancer, or who require an implant. Therefore, women with PHI are more likely to be interested in health issues and medical events, and are more likely to undergo breast screening.

Of the health behavioral risk factors, smoking status was shown to be significantly associated with breast cancer screening by multivariate logistic regression after adjusting for other factors. Other studies have shown that being a nonsmoker is a significant predictor of annual participation in a breast cancer screening program [28], possibly reflecting an increased knowledge about the negative health effects of smoking. Many studies have also reported differences between smokers and nonsmokers in psychosocial variables that seem to influence health-behavior decisions [32,33]. The Korean Ministry for Health, Welfare and Family Affairs (KMIHWAF) also supports national interventions for smoking cessation by providing public health services. To improve health behavior, including breast screening, it is important to emphasize the importance of breast screening while also recommending smoking cessation to smokers receiving these services.

In our study, women with a negative attitude towards the effectiveness of medical evaluation for early detection were less likely to undergo breast screening than those with a positive attitude. Increases in the breast cancer screening rate in the United States are probably due to changes in attitudes to mammography through appropriate education [34]. Our results show that the attitude towards medical evaluation for early detection is closely associated with the breast cancer screening rate; hence, education and public campaigns regarding the importance of cancer screening for early detection including breast cancer screening are needed to induce changes in attitude and, ultimately, to increase the breast cancer screening rate.

Factors associated with breast cancer screening program participation may differ between developed and developing countries. Recently, the breast cancer screening rate has increased to 70% in the United States. Many studies have suggested that, in the United States, having access to a physician who recommended mammography was the strongest predictor of breast cancer screening, whereas breast cancer awareness campaigns and socio-economic barriers such as low income, unemployment and a low education level, were less important in predicting breast cancer screening [35-39]. However, in Korea, the breast cancer screening rate is still low (10-50%), indicating that Korean women are not yet fully aware of the importance of breast cancer screening. Special attention should be paid to the elderly, those with a low education level, smokers, and those with a negative attitude towards the effectiveness of a medical examination or who have not previously undergone a medical examination. Education and health campaigns should be used to inform these individuals of the benefits of breast cancer screening; the participation rate in Korea may then reach that of the United States, where socio-demographic factors such as education have a smaller effect on breast cancer screening rates.

Our study also has several limitations. First, the findings were based on patient self-reported health status data and may, therefore, suffer from some inaccuracy due to respondents giving inaccurate reports. Second, Information about breast cancer screening was obtained from the responses to a single question, and any symptoms at the time of the examination were not reported, although cancer screening prevention programs of Korea are designed for individuals with no associated symptoms; therefore, there might be some misclassification of breast cancer screening participation by including individuals with symptoms indicative of breast cancer. However, several previous studies have also not considered accompanying symptoms, and used a similar definition of cancer screening participation [28,40,41]. Third, depression was also assessed by the self-report questionnaire rather than being diagnosed by a doctor, so it cannot be said to accurately indicate the incidence of medical depression. Fourth, because our study used the previous national survey, we could not collect detailed information about risk factors of breast cancer or screening specific variables such as history of breast feeding or parity, and occupational physical activity.

## Conclusions

We found that less than one-third of Korean women aged ≥ 40 years complied with breast cancer screening recommendations. To improve the participation rate for breast cancer screening, more attention should be given to vulnerable individuals, especially women of advanced age or with low education levels. In addition, our study indicates that increased public education and promotional campaigns regarding the effectiveness of medical evaluation for early detection, including breast cancer screening, are needed to increase participation in breast cancer screening programs.

## Competing interests

The authors declare that they have no competing interests.

## Authors' contributions

KL participated in the design of the study and drafted the manuscript. HL performed the statistical analysis and drafted the manuscript. SP participated in the design of the study and helped to draft the manuscript. All authors read and approved the final manuscript.

## Pre-publication history

The pre-publication history for this paper can be accessed here:

http://www.biomedcentral.com/1471-2407/10/144/prepub
